# Survey data to identify the selection criteria used by breeders of four strains of Pakistani beetal goats

**DOI:** 10.1016/j.dib.2020.106051

**Published:** 2020-07-20

**Authors:** Faisal Ramzan, Muhammad Sajjad Khan, Shaukat Ali Bhatti, Mehmet Gültas, Armin O. Schmitt

**Affiliations:** a Breeding Informatics Group, Department of Animal Sciences, Georg-August University, Margarethe von Wrangell-Weg 7, 37075 Göttingen, Germany; b Institute of Animal and Dairy Sciences, University of Agriculture Faisalabad, 38000 Faisalabad, Pakistan; c Center for Integrated Breeding Research (CiBreed), Georg-August University, Albrecht-Thaer-Weg 3, 37075 Göttingen, Germany

**Keywords:** Selection criteria, Beetal goats, Strains, Participatory approach, Survey questionnaire, Ranking

## Abstract

This article presents raw data from a survey conducted to identify the selection criteria of breeders raising either of four strains of Beetal goats, namely Beetal Faisalabadi, Beetal Makhi-Cheeni, Beetal Nuqri, and Beetal Rahim Yar Khan. After a pre-survey, a questionnaire was developed and a survey was conducted at four sites of the Punjab province of Pakistan: Faisalabad/Sahiwal, Bahawalpur/Bahawalnagar, Rajanpur, and Rahim Yar Khan. Each of these sites was the home tract of one strain. During the survey breeders (*n* = 162) were asked to rank the traits of their selection criteria based on the relative importance of those traits. Furthermore, the prevailing production system was also characterized by the breeders. For the interpretation of the results of this survey the readers are referred to Ref. [1]. The raw data set provided in this article can be extended in the future to include more strains of Beetal goats as well as other goat breeds. The selection criteria of breeders can change over time. This data set can also be used in future studies to investigate the temporal changes in the relative importance of different traits for the breeders. The factors potentially influencing those changes can also be investigated. This data set can further be utilized to design community based breeding plans tailored to the needs of the goat farming community.

Specifications table

**Table:** 

Subject	Animal Science and Zoology
Specific subject area	Animal Breeding
Type of data	Table
How data were acquired	Survey based on a researcher-made questionnaire
Data format	Raw survey data
Parameters for data collection	Experienced and highly reputed goat breeders were identified for this survey who were rearing only true bred animals and were selecting goats based on some specific criteria.
Description of data collection	A questionnaire containing a list of goat traits was designed in a pre-survey and in the actual survey these traits were ranked by the goat breeders. Some socio-economic and production characteristics of breeders were also recorded. The questionnaire used in this survey is given in Supplementary file 1. The data of socio-economic and production characteristics are given in Supplementary file 2. Each row in this file represents a breeder with the name of the goat strain given in the first column. The following columns contain the characteristics of breeders recorded in this survey. Supplementary files 3 and 4 contain the ranks given to doe and buck traits, respectively. In these files as well, each row represents a breeder with the name of the strain given in the first column while each of the other columns contains ranks given to a trait by the breeders.
Data source location	The districts of Bahawalpur (29.23° N, 71.41° E, 181 m above sea level (m.a.s.l.)), Bahawalnagar (29.59° N, 73.15° E, 163 m.a.s.l.), Faisalabad (31.25° N, 73.52° E 183 m.a.s.l.), Sahiwal (30.39° N, 73.63° E, 152 m.a.s.l.), Rajanpur (29.61° N, 70.19° E, 97 m.a.s.l.), and Rahim Yar Khan (28.25° N, 70.18° E, 80 m.a.s.l.) in the Punjab province of Pakistan.
Data accessibility	Data are available in this article as three supplementary files along with the questionnaire used in the survey.
Related research article	[1] Ramzan, F., Khan, M.S., Bhatti, S.A., Gültas, M. and Schmitt, A.O., 2020. Breeding objectives and selection criteria for four strains of Pakistani Beetal goats identified in a participatory approach. Small Ruminant Research, p.106163. https://doi.org/10.1016/j.smallrumres.2020.106163

**Value of the data**

• These data present information on the traits that constitute the selection criteria used by breeders of different strains of the Beetal goats. The ranks of these traits provide their relative importance in the respective production systems. The information on some of the basic production and socio-economic characteristics is also provided which can help understand the reasons of these breeders’ preferences.
• The data show the preference for the morphological and subjective traits over production traits amongst the Beetal breeders. These data, on the one hand show the heterogeneity of selection criteria amongst different strains while, on the other hand, also show the inclination of breeders keeping the same strain to prefer similar traits.
• Beetal breed is the third most abundant breed in Pakistan. The importance of Beetal goats has increased over the years mainly due to their higher meat and milk production potential. Currently, many projects focus on improving the genetic potential of this breed. In this regard, this data can be useful to those working on topics like understanding different decision-making processes of goat breeders. Furthermore, this data can be useful to design community level breeding plans to enhance the proper utilization of the genetic resources of goats.
• The precise knowledge about the population size of the different Beetal strains is limited. With the availability of this knowledge in the future, this dataset can be updated and completed with more strains. The extended data can also be used in future surveys to elaborate the changing preferences of the breeders over time. These changes in the preference for different traits amongst the breeders can change the utility of animals in the given production system. Better understanding of these changing preferences can help design a breeding program tailored to meet future needs.

## 1 Data description

We present three files containing the raw data obtained in our survey. Each file contains all four strains under sinvestigation. The questionnaire used in this survey is also provided (Supplementary File 1). The socio-economic and production characteristics data which were originally categorical were recoded to represent each category with a label. For example, to record the education level of breeders categories like primary, secondary, higher secondary or no education were given in the questionnaire which were later labelled using consecutive numbers from 1 to 4, respectively. More information on the categories and their labels is provided along with the data file (Supplementary File 2). The other files contain the traits as variables with their ranks as assigned by the breeders according to their relative importance in the selection of does (Supplementary file 3) and bucks (Supplementary file 4). There are 18 and 14 traits that constitute the selections criteria for does and bucks, respectively. The highest rank (most preferred trait) given in this data is 1 while the lowest rank (worst rank) is 7 for does and 6 for buck traits. The traits that were not ranked by the breeder were given the rank 0 which represents that the breeder was not selecting for that trait.

## 2 Experimental design, materials, and methods

The survey was conducted at four sites that were the home tract (region having a higher concentration of a strain) of the four Beetal strains in the Punjab province of Pakistan. Each site constituted either one or multiple districts which were the home tract (region having a higher concentration of a strain) of one of the four strains of Beetal goats considered in this survey. For the description of the Beetal breed, its different strains and their home tracts readers are referred to Ref. [2]. To choose a survey methodology that is best comprehensible to the Beetal breeders, a pre-survey was conducted before the actual survey. For this survey only those goat farmers were chosen who, unlike most farmers, were rearing only true bred animals and were selecting goats based on some specific criteria. Those farmers were regarded as goat breeders. In the pre-survey, breeders were asked to provide a list of the most important goat traits. Then a questionnaire was developed that contained all those traits. Furthermore, a ranking methodology was found to be most suitable for the breeders and was used for our actual survey. For the survey, a total of 162 Beetal goat breeders were interviewed. Along with aforementioned criteria, more experienced and highly reputed breeders were chosen for the study with the help of local extension workers of the Livestock and Dairy Development Department of Punjab. From the above-mentioned sites 33 Beetal Makhi-Cheeni, 41 Beetal Faisalabadi, 43 Beetal Nuqri and 45 Beetal Rahim Yar Khan breeders participated in the survey. The same numerator conducted all interviews with the help of facilitators to help translate terms in the local languages. In the interviews, breeders were asked to enlist the traits for which they would select their goats as parents of the next generation followed by the ranking of those traits based on their relative importance. The ranking was ordinal, with rank 1 as the highest rank. The breeders could allocate as many ranks as there were traits which were relevant to them and they did not have to rank all the traits present on the questionnaire. Furthermore, if some preferred traits were not available on the given questionnaire, the breeders could add those traits to the list and rank them as well. They could allocate the same rank to multiple traits as well if those carried the same importance for the breeders. Some socio-economic and production characteristics of breeders were also recorded. The age, education level, ownership of agricultural land, herd size, source of replacement does and bucks and different selling stages of the goats were documented.

## Ethics statement

Since this was a non-experimental, voluntary survey, no ethical approval was required.

## Declaration of Competing Interest

The authors declare that they have no known competing financial interests or personal relationships which have, or could be perceived to have, influenced the work reported in this article.

## Appendix Supplementary materials

Image, application 1

Image, application 2

Image, application 3

Image, application 4

Image, application 5
