# Supplementary material for: Survey data to identify the selection criteria used by breeders of four strains of Pakistani beetal goats
Source: Data Brief. 2020 Jul 20;32:106051. doi: 10.1016/j.dib.2020.106051 (PMC7403874; doi:10.1016/j.dib.2020.106051)
Supplement: Supplementary file 2 [file mmc2.docx]

**Questionnaire**

BREEDING OBJECTIVES FOR BEETAL GOAT

Date: _____________________Breeder Name: ________________________________________

Place of survey: ______________________________Mobile No.: _________________________

Age: _______________________ Education: _________________________________________

Address: _______________________________________________________________________

Number of goats: ________________ Strain: __________________________________________

|  | **Source of feed:** | a) Grazing b) Fodder c) Both fodder and grazing |
| --- | --- | --- |
|  | **Land owned:** | a) Yes b) No |
|  | **Source of Bucks:** | a) Indigenous to flock b) Purchase c) Both a&b d) Other |
|  | **Source of Does:** | a) Indigenous to flock b) Purchase c) Both a&b d) Other |
|  | **Males sold as:** | a) Kids b) Bucks c) Castrated males on Eid d) All e) ….&….. |
|  | **Females sold as:** | a) Kids b) Yearlings c) Does d) All e) …. &.... f) None |

**Rank the following traits on the basis of their relative economic importance:**

| Traits of doe | Ranks | Traits of Buck | Ranks |
| --- | --- | --- | --- |
| (1) Coat color |  | (1) Coat color |  |
| (2) Horns |  | (2) Horns |  |
| (3) Nose shape |  | (3) Nose shape |  |
| (4) Ear size |  | (4) Ear size |  |
| (5) Neck length |  | (5) Neck length |  |
| (6) Skin |  | (6) Skin |  |
| (7) Purity |  | (7) Purity |  |
| (8) Tail length |  | (8) Tail length |  |
| (9) Eye color |  | (9) Eye color |  |
| (10) Pole width |  | (10) Pole width |  |
| (11) Body length |  | (11) Body length |  |
| (12) Body height |  | (12) Body height |  |
| (13) Milk yield |  | (13) Testicular size |  |
| (14) Udder size |  | (14) Testicular shape |  |
| (15) Teats size |  | (15) Legs’ strength |  |
| (16) Udder shape |  | (16) Color of the kids |  |
| (17) Twining ability |  | (17) Kids’ performance |  |
| (18) Legs’ strength |  | (18) Dam’s performance |  |
| (19) Kids’ performance |  |  |  |
| (20) Dam’s performance |  |  |  |
